# Supplementary material for: Are carbon emissions trading and green financial instruments synergistic? -Comprehensive quantitative research based on content analysis
Source: PLoS One. 2024 Mar 7;19(3):e0298601. doi: 10.1371/journal.pone.0298601 (PMC10919601; doi:10.1371/journal.pone.0298601)
Supplement: S1 Data — (ZIP) [file pone.0298601.s001.zip › Data/地级市Z_F工作报告.zip!/╔╜╢1⁄2/▒⌡╓▌╩╨/└┤╫╘╣1⁄2╓┌║┼í╛┬φ┐╦╩2╛▌═°í┐_2007.docx]

各位代表:

　　现在,我代表市人民政府,向大会作政府工作报告,请予审议,并请市政协委员和其他列席的同志提出意见。

　　一、2006年政府工作回顾

　　过去的一年,在省委、省政府和市委的坚强领导下,市政府团结依靠全市人民,以邓小平理论和“三个代表”重要思想为指导,以科学发展观统领全局,坚持“30字”工作方针,“锁定目标争上游,全力冲刺2006”,完成了市八届人大四次会议确定的各项任务,实现了“十一五”的良好开局。全市实现生产总值829.02亿元,增长17.4%;地方财政收入45.12亿元,增长41.3%;社会消费品零售总额208.57亿元,增长16.1%;城市居民人均可支配收入11726元,农民人均纯收入4370元,分别增长14.0%和14.5%。

　　(一)不断调整优化结构,经济增长质量明显提高。一、二、三产业结构继续优化,三产比例达到11.7:62.1:26.2。实现农业总产值180.33亿元,增长5.1%;全部免除了农业税,减轻农民负担1.9亿元,人均67元。全力打造十大产业(链)集群,规模以上工业实现增加值443.79亿元、利税157.96亿元、利润90.94亿元,分别增长28.6%、38.8%和41.1%;规模以上企业达到1097家,其中魏桥创业集团销售收入突破500亿元大关,滨化、鲁北集团销售收入超过100亿元。新增中国名牌3个、中国驰名商标2个。旅游、物流等第三产业发展迅速,实现服务业增加值216.99亿元,增长20.3%。高新技术产业产值达到168.74亿元,增长39.3%。具有自主知识产权的“滨州芯片”研发成功,顺利实现了汽车上路、轮船下海、飞机批量生产。民营经济实缴税金47.5亿元,增长33.4%,注册私营企业已达7161户。县域经济协调快速发展,新增5个省级开发区,实现了县区都有省级开发区的目标。招商引资实际利用市外资金462.83亿元,其中实际外商直接投资1.52亿美元,增长62.6%。实现进出口总额27.03亿美元,增长20.4%,其中出口15.83亿美元,增长27.5%。金融机构各项存、贷款分别比年初增加76.06亿元和121.41亿元。第四届银企合作洽谈会签约资金到位460亿元,第五届银企合作洽谈会贷款金额达718亿元。

　　(二)快速推进基础设施建设,支撑发展的能力显著增强。全面落实国家宏观调控政策,完成规模以上固定资产投资469.71亿元,增长19.7%。黄河公铁大桥胜利合龙,滨州铁路一期工程主线全线完工。津汕、威乌高速公路滨州段实现路基贯通,滨德高速公路通过土地评审。大济路40公里一级路建成通车,干线公路改造工程全部完成。完成农村公路新建、改造1352公里。惠青黄河大桥建成通车,渤海大桥、滨港供水工程奠基开工。滨州万吨港建设全面展开,导堤跨过拦门沙。“北带”开发进程加快,续建和新建防潮堤79公里。临港经济开始起步,5家造船项目落户滨州。邮政通信发展迅猛。新一轮城市总体规划通过省政府审查,全市控制性详细规划覆盖率达80%。市区城市基础设施建设投资10.48亿元,相继完成了一批道路、绿化、公交、供热、供气、供水、污水处理等公用设施工程,城市功能进一步增强。秦台干沟市区段治理全部完成,张肖堂干渠治理市区段完成70%。四环五海、36桥、72湖建设快速推进,中海被命名为“国家水利风景区”。大力实施城市环境综合整治,执法管理迈上新台阶。滨州入选中国魅力城市200强。

　　(三)加强统筹协调,各项社会事业全面发展。大力推进科教、人才建设,科学支出及科技三项费用达到1.13亿元,增长61.0%,引进院士、博士、硕士人才307名。义务教育保障能力进一步增强,推进素质教育,教育教学质量稳步提高,普通高考再创历史新高;滨州市技术学院申办成功;农村中小学危房改造竣工12.2万平方米。现代吕剧《梨花雨》荣获全国第三届少数民族文艺汇演创作金奖,市图书馆、艺术馆迁至国际会展中心。成功举办了首届国际通用航空产业博览会、第四届国际家纺文化节等一系列重大节会活动。我市运动员在15届亚运会和21届省运会上分别取得2枚和64枚金牌的优异成绩,全民健身运动深入开展。公共卫生“两个体系”建设16个项目全部完成。顺利通过国家卫生城市省复核验收。人口与计生综合改革不断深化,全面完成了年度人口责任目标。社会保障体系不断完善,“两个确保”继续得到巩固。国有粮食购销企业全部完成改革任务,事业单位改革启动试点,农村综合改革扎实推进。创建全国双拥模范城工作成效显著,军政军民团结进一步增强。广播电视、民族宗教、新闻出版、外事侨务、对台、统计、物价、老龄、档案、史志、地名、地震、气象、海事、人防、油区管理、民兵预备役、国防动员等工作都有了新的发展。

　　(四)关心关注群众生产生活,十大民心工程顺利完成。(1)帮扶阳信、惠民工作取得突破性进展,成效显著。(2)新农村建设编制了300个中心村的指导性规划,对190个村进行了环境综合整治,100个新农村示范村呈现新面貌。(3)组织开展了资源节约、环境保护“百日会战”,节能降耗取得明显成效,二氧化硫和化学需氧量排放量分别下降5.7%和4.2%。市内小流域控制断面水质稳定改善,城区空气质量良好率达到95.9%,林木覆盖率达到22.7%。(4)城镇新增就业再就业4.64万人,新增劳务输出6.5万人,城镇登记失业率2.9%。(5)新增蓄水能力6935万立方米,新增农村通自来水人口53.14万人,农村自来水普及率达到83.6%,提前完成了省下达任务。(6)博兴、无棣列为第四批新型农村合作医疗省级试点县,沾化列入市级试点,全市参合农民达162.19万人,参合率89.51%,受益面104.47万人次;完成22家乡镇卫生院改造。(7)健全完善社会救助体系,出台了城乡救助十个暂行办法,完成76处乡镇敬老院建设任务,集中供养能力达到70%;提高低保标准,为5.31万城乡低保人口发放低保金3408万元,实现应保尽保;为困难学生发放“两免一补”资金2100万元。(8)对特困大学生实施救助,发放救助金130万元。(9)探索建立被征地农民保障机制,采用“一卡通”直接兑付征地补偿。(10)开工建设经济适用房50万平方米,安置“双困”家庭2680户,廉租住房制度建设稳步推进。

　　(五)大力营造良好发展环境,政府管理和公共服务水平不断提高。自觉接受市人大及其常委会的监督,加强同市政协的联系,认真办理了市人大常委会审议及视察意见、人大代表建议和政协委员提案。市公共行政审批服务中心受理办结审批项目8.25万件,符合条件的审批事项按时办结率100%;市长公开电话中心受理群众来话6.15万件,办复率99.7%;规范政府采购管理,强化审计工作,提高了资金使用效益。深入开展廉政建设和反腐败斗争,狠抓商业贿赂专项治理,严肃查处违法违纪案件。积极开展联合大接访、“民主议政日”活动,狠抓社会治安综合治理,落实安全生产责任制,大力推进“平安滨州”建设,全市上下和谐稳定。

　　各位代表!过去一年的发展成就,是省委、省政府和市委正确领导的结果,是市人大、政协和社会各界监督、关心、支持的结果,是全市人民同心同德、艰苦奋斗的结果。在此,我代表市政府,向全市人民,向各位人大代表、政协委员,向各民主党派、工商联、各人民团体和各界人士,向驻滨人民解放军、武警官兵和公安干警,向所有关心支持滨州发展的海内外各界朋友,表示衷心的感谢!

　　同时,我们也要清醒地看到,全市经济总量不大、整体实力不足、结构不合理、社会发展与经济发展不够协调的局面还没有根本改变;经济增长方式粗放、创新能力不强、南北发展不平衡的矛盾仍然比较突出;环境保护、节能降耗、新农村建设任务还十分艰巨;政府职能转变不到位,公共服务体系建设滞后,社会管理、公共服务水平不够高,执法体系不够完善的问题还在不断显现;观念不新、效率不高、作风不实、官僚主义、推诿扯皮,甚至以权谋私、贪污腐败等现象在政府机关和公务员队伍中还依然存在。对这些问题,我们必须高度重视,采取得力措施,认真加以解决。

　　二、2007年工作的总体要求和目标任务

　　“奋力拼搏2007,科学发展再创佳绩”。综合分析国际国内经济发展的大趋势,我们既面临着良好的发展机遇,也面临着严峻的挑战。根据中央、省委、省政府和市委的部署,今年的政府工作,要以邓小平理论和“三个代表”重要思想为指导,以科学发展观总揽全局,认真贯彻党的十六大、十六届三中、四中、五中、六中全会和中央、省经济工作会议精神,坚决执行“30字”工作方针,全面实现“12345”的目标要求,即坚持科学发展“一个主题”,锁定经济总量巩固全省中游、人均指标进入全省上游“两个目标”,突出经济、和谐社会、执政能力“三大建设”,实施招商、高新、三产、港铁“四个拉动”,确保地方财政收入、城镇居民收入、农民现金收入、城乡就业率、群众消费水平“五个提高”,全力推进经济社会又好又快发展,加快建设民富市强、和谐文明的现代滨州。今年主要预期目标是:完成生产总值970亿元,增长17%;规模以上工业增加值555亿元,增长25%;全社会固定资产投资720亿元,增长20%;地方财政收入56亿元,增长25%。主要约束目标:万元GDP能耗降低4.5%;化学需氧量排放量降低4.6%;二氧化硫排放量降低23.4%;人口自然增长率控制在6.6‰。

　　为实现上述目标任务,在工作指导上把握好以下五个方面:一是必须坚持科学发展、加快发展。要正确处理“好”与“快”的关系,明确发展定位,提升发展标准,超越发展的决心不动摇,有效益的增长不减速。二是必须坚持党的领导。时刻与党中央和省委、市委保持高度一致,毫不动摇地贯彻执行市委的重大决策和部署,增强党性、悟性、可靠性,提高执行力,全市一盘棋,狠抓各项工作落实。三是必须坚持重点突破。从实际出发,突出工作重点,选准突破口,有所为有所不为,集中人力、物力、财力办大事。四是必须坚持执政为民。千方百计为群众办好事、办实事,让人民群众在发展中得到更多实惠,努力促进和谐社会建设。五是必须坚持开拓创新。把做好今年工作与实现“十一五”规划结合起来,不断增强工作的前瞻性、预见性和主动性,全力推进经济社会发展实现新突破。

　　三、全面落实科学发展观,推动经济又好又快发展

　　紧紧抓住发展第一要务,转变发展观念,创新发展模式,提高发展质量,着力实施“五个加快推进”。

　　(一)加快推进社会主义新农村建设。坚持工业反哺农业、城市支持农村和多予少取放活的方针,促进“三农”工作全面发展。完善农村投入稳定增长机制,市级财政安排新农村建设资金9780万元,增长52.6%。认真执行粮食生产各项扶持政策,落实最严格的耕地保护制度。大力发展现代农业、特色农业,农业标准化基地面积达到150万亩,市级龙头企业达到150家。搞好重大畜禽疫病防控、农技推广和农业机械化建设,培育发展各类农村经济协会。抓好农田林网、公路、村庄绿化、防护林带和高酸苹果基地建设,完成造林折实面积20万亩。加快农村基础设施建设,重点抓好200个专项示范村建设,完成100个中心村的村容村貌整治。新增蓄水能力6000万方,解决和改善46万人的饮水困难问题,农村自来水普及率达到95%以上。完善农村远程教育网络和有线电视村村通工程。新建、改造农村公路700公里。从今年起免除农村义务教育阶段学生杂费,对农村义务教育阶段学生和城镇家庭贫困学生实行“两免一补”(免杂费、免书本费、补助寄宿生生活费)。大力培养新型农民,每年培训农民10万人以上。

　　(二)加快推进经济增长方式转变。一是大力实施“工业兴市”战略。积极开展“产业链延伸年”活动,围绕十大产业集群,突出研发、配套、资金、市场、管理、信息、环保、效益八个环节,狠抓高新项目、支柱产业、骨干企业、品牌产品,做到延伸有项目、项目有人抓、考核有人管。对20家大企业领办的工业园和47家成长型中小企业进行重点扶持,快速推进产业升级,加快形成产业集群和特色优势,促进规模、质量、效益大幅提高。规模以上工业主营业务收入力争实现2100亿元,增长27%。二是大力发展现代服务业。突出发展金融保险、现代物流、旅游、中介、会展、商贸等产业。加快大企业的主辅分离、制造业和服务业分离。规划建设“鲁北大街”,壮大专业市场,形成服务业发展的样板带。确保第五届银企合作洽谈会贷款资金到位650亿元,办好第六届银企合作洽谈会。争创中国优秀旅游城市,年内力争实现旅游总收入23.4亿元。三是大力发展民营经济。认真落实各项扶持政策,促进民营经济铺天盖地发展。新发展3家担保公司,信用民营企业达到260家,民营经济实缴税金力争达到56亿元。四是大力发展高新技术产业。开展“航空发展年”活动,围绕汽车、船舶、飞机的制造、组装与销售及零部件配套等,大力发展先进装备制造业。在电子信息产业、光伏太阳能、数字机顶盒、生物医药、新材料等产业和项目上取得新进展。新认定省级高新技术企业10家以上,省级工程技术中心2家以上。高新技术产业产值力争突破200亿元。五是大力推进名牌战略。围绕延伸壮大“十大产业链”,争创中国名牌1-2个、中国“驰名商标”1个,国家免检产品2—3个;山东名牌10个,山东“著名商标”5个。

　　(三)加快推进重大基础设施建设。加快以两路(高速公路、铁路)、两港(海港、空港)为重点的基础设施建设。威乌、津汕高速公路滨州段年内竣工通车。滨德高速公路、孙武黄河大桥力争年内开工。加快滨州西客站等场站建设,全面提高营运能力。落实“铁路运营年”部署要求,滨港铁路一期工程实现运营,火车客运站、滨北货运站、沾化火车站力争年底建成;做好滨港铁路二期规划设计和黄大铁路、德滨铁路前期工作。以“港口建设年”为契机,按照“航道疏浚、商港、造船、渔港、油盐化工”五位一体和“航道疏浚、西导堤、渤海大桥、套尔河大桥、套尔河沿岸码头”五路并进的思路,加快建设现代化万吨大港,膨胀发展临港产业。加快推进滨港供水工程以及鲁北生态电厂、沾化电厂、国电博兴电厂等项目建设。搞好城市规划建设,强化城乡环境综合整治,加大城市执法力度,提升管理水平。抓好市区骨干道路、排污、环卫、绿化、美化、亮化、交通管理等基础设施建设;城区新增集中供热面积50万平方米,新增天然气日供气量2万立方米。开工建设20万平方米经济适用房,解决2000户居民住房问题。

　　(四)加快推进资源节约型、环境友好型社会建设。大力开展“节能环保绿化年”活动,努力实现可持续发展。一是深入推进节能降耗。突出抓好56户重点企业的节能工作,鼓励发展新型能源,推广节能技术,淘汰落后设备和工艺。二是切实加强环境保护。以全面落实“十一五”环保目标责任书年度任务为主线,加快创建国家环保模范城市步伐,深入实施“蓝天碧水”工程,突出热电企业脱硫工程、污水处理厂建设运营,严格环评和“三同时”制度,努力改善环境质量。三是大力发展循环经济。推广鲁北、西王经验,年内新培育10个循环经济试点企业、3个试点园区。在重点区域、行业、企业和生产、流通、消费各个环节,积极推广能源综合利用先进技术。四是加快生态市建设。抓好小清河流域和潮河等六河流域的综合整治,对中海、贝壳堤岛与湿地自然保护区和南部山区等生态自然景观进行升级改造。强力推进黄河标准化堤防建设,实施张肖堂干渠市区段综合治理,搞好新立河等水利风景区配套开发。

　　(五)加快推进改革开放。加快国有工业和商贸流通企业改革改制步伐,建立健全现代企业制度和国有资产监管制度。加快困难企业破产重组,兑付职工权益,启动有效资产。积极推进企业市场融资,力争年内2—3家企业实现上市融资或发行债券。全面推进事业单位改革,加快经营性事业单位股份制改造。巩固农村税费改革成果,继续深化乡镇机构、农村义务教育经费保障机制和县乡财政体制改革。大力实施对外开放,创新招商方式,抓好20个利用外资过千万美元的大项目。优化出口产品结构,促进魏桥、亚光等20家重点企业对外贸易稳定增长。积极推进劳务输出和境外办厂经营。深入推进北带开发和渡河战役,进一步加大帮扶力度,继续全力突破阳信、惠民,促进南北协调发展。加快与济南都市圈、环渤海经济圈、半岛城市群和天津滨海新区的产业对接融合,抢抓黄河三角洲开发、海上山东建设的机遇,借势发展、借力突破。

　　四、繁荣发展社会事业,加快建设和谐滨州

　　坚持发展为了人民、发展依靠人民、发展成果由人民共享,突出关系群众切身利益的问题,着力抓好“五个更加注重”。

　　(一)更加注重自主创新,强化科技人才支撑。深入开展“科技进步年”活动,强力实施科教兴市、人才强市战略,加快创新型城市建设,积极申办设立省级高新技术开发区。全年申请专利400件以上,组织科技示范工程300项以上,引进高科技成果100项。落实农村义务教育“以县为主”管理体制,实现城乡教师工资同工同酬。大力推进素质教育,强化高等教育内涵建设,提高滨州学院、技术学院办学水平,滨州职业学院创建全国百强职业学院。着力建设行政人才、企业经营管理人才和专业技术人才三支队伍,加快培养和引进“十大产业(链)集群”发展急需的各类高层次人才;组织实施“金蓝领”培训工程,继续开展首席技师评选活动,培训民营企业经营管理人才1800人以上。

　　(二)更加注重协调发展,加快繁荣文体卫生事业。加大对文化体育设施的投入,鼓励社会力量捐助和兴办公益性文化体育事业。繁荣文艺创作,完善市图书馆、艺术馆等文化设施管理经营,加快建设市博物馆的前期工作,提升城市的文化功能。广泛开展群众体育活动,积极承办国际国内重大赛事,加快全国十一运会场馆建设进度。建立覆盖城乡居民的基本卫生保健制度,提高疾病预防控制和突发公共卫生事件处置能力。所有县区全面推行新型农村合作医疗试点;新改造乡镇卫生院15家,全面完成改造任务;对部分村级卫生室进行重点改造,乡村卫生一体化规范管理覆盖面达到100%,建立健全城市社区卫生服务体系。落实计划生育基本国策,确保圆满完成省下达任务指标。进一步巩固发展国家卫生城市成果。

　　(三)更加注重以人为本,健全完善社会保障体系。深入开展“解决城乡家庭零就业年”活动,新增就业再就业3万人,城镇登记失业率控制在3.8%以内,新增农村劳动力转移就业5万人。严格实施农民工工资支付保证金制度,严厉查处恶意拖欠工资行为。多渠道筹措社保基金,完善监管制度,确保基金安全和保值增值。完善城镇职工基本养老、医疗、失业、工伤和生育保险制度,建立完善农村社会养老保险制度,开展被征地农民养老保险制度试点。全面落实城乡居民最低生活保障、农村五保供养、高龄特困老年人救助等十大救助办法。城乡低保确保动态管理下的应保尽保;农村五保全面落实财政为主供养的长效机制;全面建立医疗救助和基金筹措机制,解决城乡群众“看病难”和因病致贫问题;确保考入本科院校的困难家庭大学生顺利入学、完成学业。加大经济适用房建设力度,全面建立廉租住房制度。

　　(四)更加注重文明和谐,切实加强民主法制建设。认真执行人大及其常委会的决议、决定,自觉接受人大的法律监督和政协的民主监督,高度重视新闻舆论监督和社会公众监督,主动听取各民主党派、工商联、无党派人士及各人民团体的意见建议,办理好人大代表建议和政协委员提案。深化完善政务公开、厂务公开和村务公开,大力推进和谐社区建设。认真实施“五五”普法规划,深化依法治理工作,健全完善法律援助救助体系。进一步加强审计、监察等专项监督。继续加强民兵预备役和国防动员工作,积极争创全国双拥模范城,加快建设渤海革命老区纪念园。大力弘扬新滨州精神,努力创建省级文明城市。高度重视油区工作,加强油地共建,努力做好新闻出版、民族宗教、老龄、妇女儿童、对台、侨务、档案、人防、气象、防震、修志、扫黄打非等工作。

　　(五)更加注重平安建设,营造稳定有序的发展环境。坚持以平安社区、平安行业、平安家庭建设为载体,深入开展“城乡平安和谐稳定年”活动。进一步建立完善县乡村三级信访网络,健全信访工作责任制和矛盾纠纷排查调处机制,及时化解各种社会矛盾。加强乡镇调解中心和司法所规范化建设,确保全市乡镇司法所全部达到省级规范化标准。完善应急管理机制,高度重视食品、药品安全,切实搞好监管。强化社会治安综合治理,加强基层基础工作,始终保持高压态势,严厉打击各种犯罪活动,切实维护社会公平和正义。严格落实安全生产责任制,实现市级安全生产综合监管应急救援指挥平台与县区、乡镇、企业联网,防止重特大事故的发生。

　　五、加强政府自身建设,全面提高行政水平

　　(一)转变职能,树立快捷高效的政府形象。认真履行经济调节、市场监管、社会管理、公共服务基本职能,推进政企、政社、政资、政事、政府与中介组织分开,更多地运用经济、法律手段调节经济活动,为各类市场主体提供良好服务。强化市场监管,深入整顿规范市场秩序。大力推进学习型政府、电子政务建设,深化行政审批制度改革,规范审批行为,提高行政效率和水平。全面推行政务公开,完善政府公报、政府网站、市长公开电话等公开渠道,保障人民群众的知情权、参与权、表达权和监督权。

　　(二)改进作风,树立求真务实的政府形象。深入学习贯彻胡锦涛总书记在中纪委七次全会上的讲话精神,认真开展“领导干部作风建设年”活动,大力弘扬勤奋好学、学以致用,心系群众、服务人民,真抓实干、务求实效,艰苦奋斗、勤俭节约,顾全大局、令行禁止,发扬民主、团结共事,秉公用权、廉洁从政,生活正派、情趣健康的新风正气。坚持深入基层,多为人民群众办具体事、办实在事。坚持少开会、开短会,讲短话、发短文,少说多干,只干不说。加强决策目标、执行责任、考核监督“三个体系”建设,完善重大事项专家咨询、集体决策、跟踪考核、责任追究机制,重大事项和重点工作都要分解细化,明确工作标准和完成时限,言必信,行必果,答应的事、承诺的事都必须给人民群众一个明确的交代。

　　(三)廉洁勤政,树立清正廉明的政府形象。全面落实党风廉政建设责任制,积极构建惩防体系和廉洁自律机制,强化领导干部经济责任审计,切实从源头上防治腐败。坚持依法行政,从严治政,强化行政执法监督,规范行政执法行为。大力发扬艰苦奋斗的精神,加快建设节约型机关,坚决反对铺张浪费。要深入治理商业贿赂,切实纠正部门和行业不正之风,严厉查处各种名目损害群众利益的行为,对任何违法违纪案件和腐败分子都要一查到底,依法严惩,决不手软。政府组成人员和广大公务员特别是主要负责人必须认真执行廉洁从政的各项规定,防微杜渐,警钟长鸣,积极践行“八荣八耻”荣辱观,自觉与各种腐败现象作坚决斗争。

　　各位代表!滨州大地正在发生着一场深刻的历史性变化,滨州人民正在超越发展的康庄大道上阔步前进!让我们高举邓小平理论和“三个代表”重要思想伟大旗帜,全面落实科学发展观,紧密团结在以胡锦涛同志为总书记的党中央周围,在省委、省政府和滨州市委的正确领导下,以更加饱满的热情、更加昂扬的斗志、更加创新的精神、更加扎实的工作,鼓足干劲,奋力拼搏,为建设民富市强、和谐文明的现代滨州而努力奋斗!
